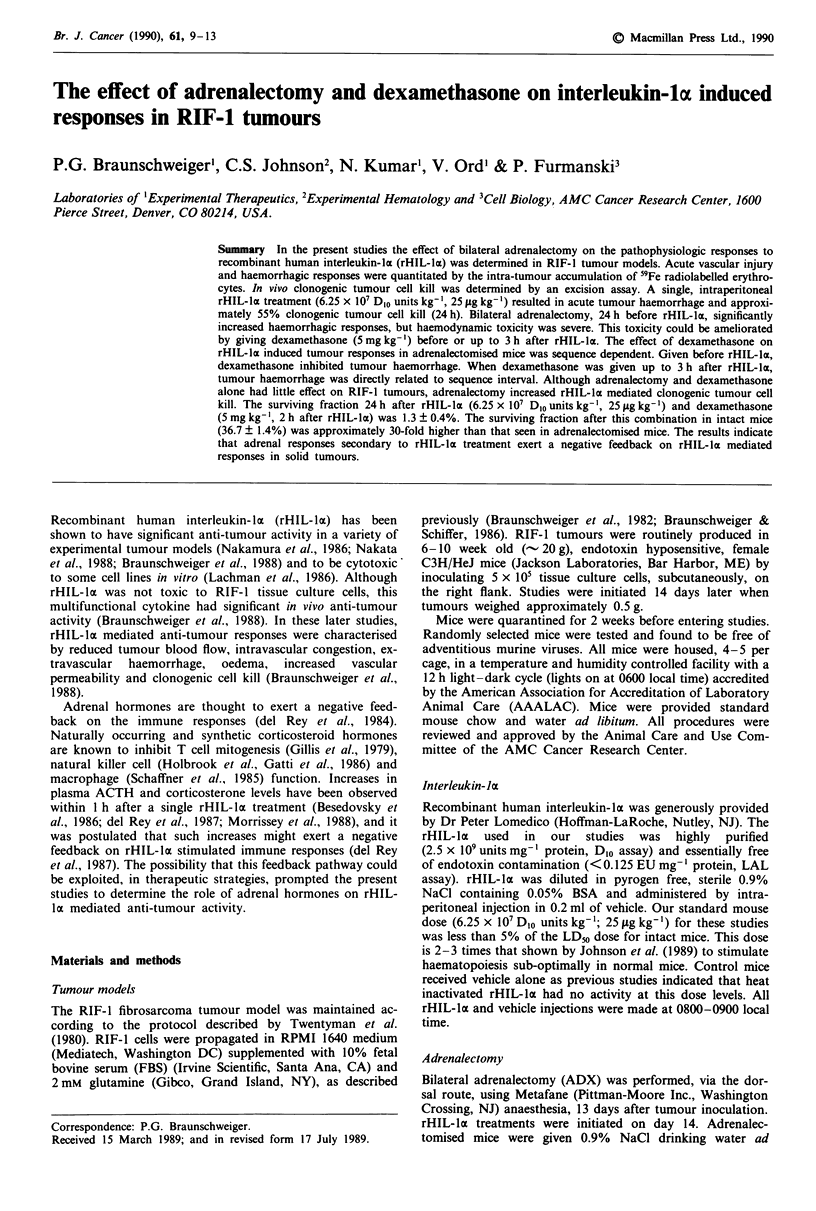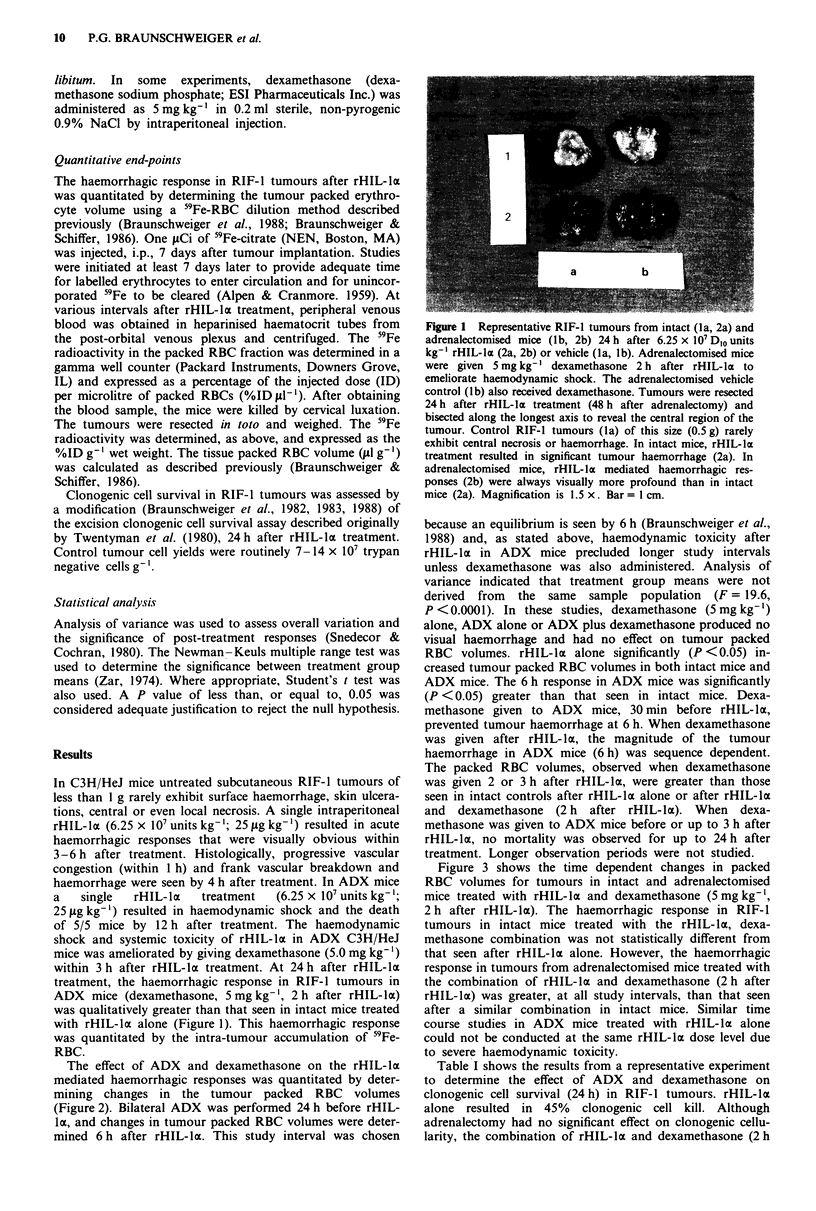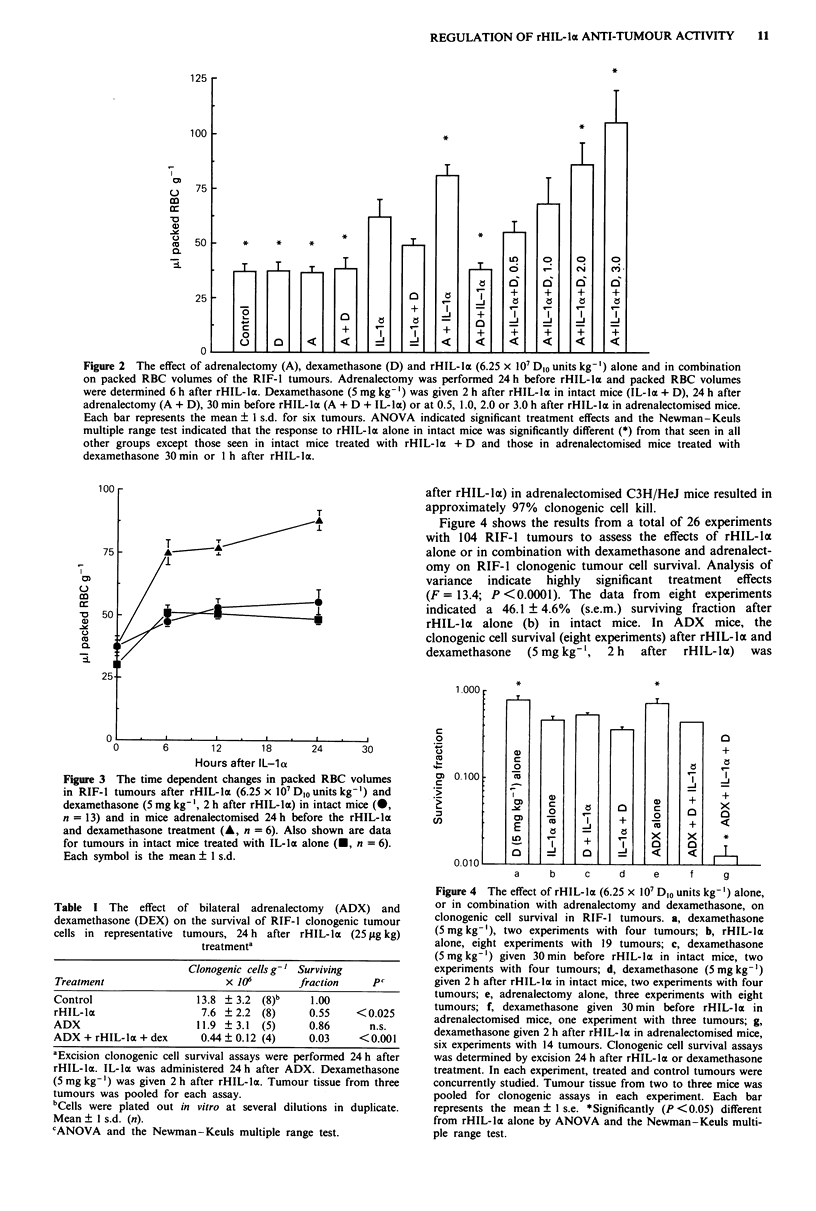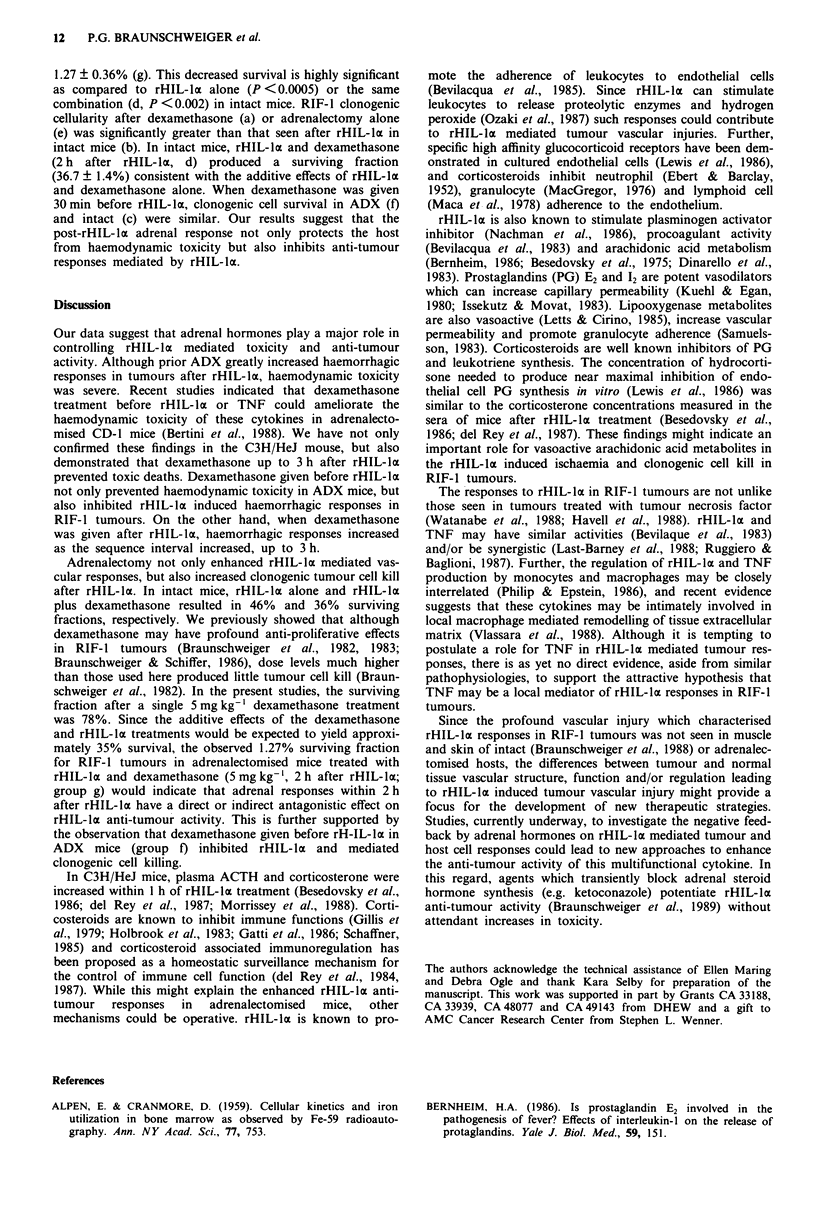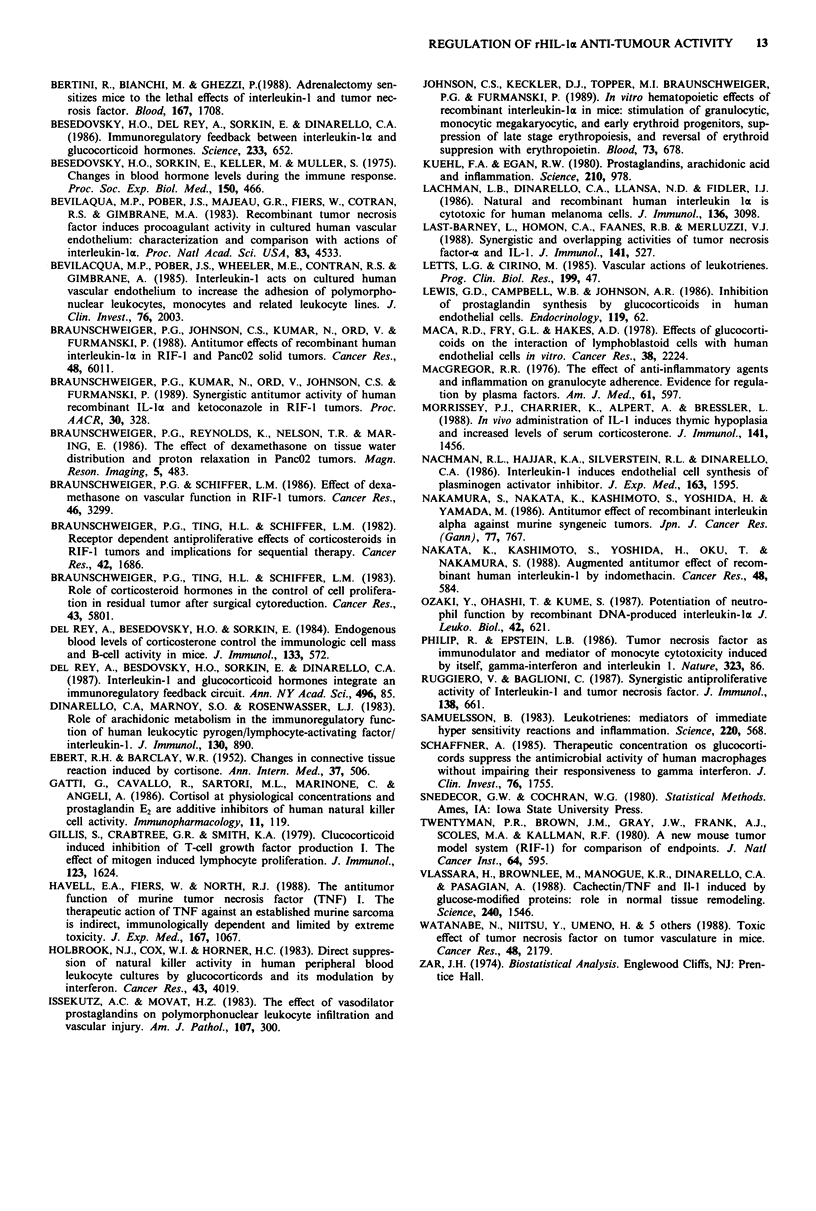# The effect of adrenalectomy and dexamethasone on interleukin-1 alpha induced responses in RIF-1 tumours.

**DOI:** 10.1038/bjc.1990.4

**Published:** 1990-01

**Authors:** P. G. Braunschweiger, C. S. Johnson, N. Kumar, V. Ord, P. Furmanski

**Affiliations:** Laboratory of Experimental Therapeutics, AMC Cancer Research Center, Denver, CO 80214.

## Abstract

**Images:**


Br. J. Cancer (1990), 61, 9-13 © Macmillan Press Ltd., 1990

### The effect of adrenalectomy and dexamethasone on interleukin-1α induced responses in RIF-1 tumours

P.G. Braunschweiger[1], C.S. Johnson[2], N. Kumar[1], V. Ord[1] & P. Furmanski[3]

*Laboratories of [1]Experimental Therapeutics, [2]Experimental Hematology and [3]Cell Biology, AMC Cancer Research Center, 1600 Pierce Street, Denver, CO 80214, USA.*

**Summary** In the present studies the effect of bilateral adrenalectomy on the pathophysiologic responses to recombinant human interleukin-1α (rHIL-1α) was determined in RIF-1 tumour models. Acute vascular injury and haemorrhagic responses were quantitated by the intra-tumour accumulation of $^{59}$Fe radiolabelled erythrocytes. *In vivo* clonogenic tumour cell kill was determined by an excision assay. A single, intraperitoneal rHIL-1α treatment ($6.25 \times 10^7$ $D_{10}$ units $kg^{-1}$, 25 μg $kg^{-1}$) resulted in acute tumour haemorrhage and approximately 55% clonogenic tumour cell kill (24 h). Bilateral adrenalectomy, 24 h before rHIL-1α, significantly increased haemorrhagic responses, but haemodynamic toxicity was severe. This toxicity could be ameliorated by giving dexamethasone (5 mg $kg^{-1}$) before or up to 3 h after rHIL-1α. The effect of dexamethasone on rHIL-1α induced tumour responses in adrenalectomised mice was sequence dependent. Given before rHIL-1α, dexamethasone inhibited tumour haemorrhage. When dexamethasone was given up to 3 h after rHIL-1α, tumour haemorrhage was directly related to sequence interval. Although adrenalectomy and dexamethasone alone had little effect on RIF-1 tumours, adrenalectomy increased rHIL-1α mediated clonogenic tumour cell kill. The surviving fraction 24 h after rHIL-1α ($6.25 \times 10^7$ $D_{10}$ units $kg^{-1}$, 25 μg $kg^{-1}$) and dexamethasone (5 mg $kg^{-1}$, 2 h after rHIL-1α) was 1.3 ± 0.4%. The surviving fraction after this combination in intact mice (36.7 ± 1.4%) was approximately 30-fold higher than that seen in adrenalectomised mice. The results indicate that adrenal responses secondary to rHIL-1α treatment exert a negative feedback on rHIL-1α mediated responses in solid tumours.

Recombinant human interleukin-1α (rHIL-1α) has been shown to have significant anti-tumour activity in a variety of experimental tumour models (Nakamura *et al.*, 1986; Nakata *et al.*, 1988; Braunschweiger *et al.*, 1988) and to be cytotoxic to some cell lines *in vitro* (Lachman *et al.*, 1986). Although rHIL-1α was not toxic to RIF-1 tissue culture cells, this multifunctional cytokine had significant *in vivo* anti-tumour activity (Braunschweiger *et al.*, 1988). In these later studies, rHIL-1α mediated anti-tumour responses were characterised by reduced tumour blood flow, intravascular congestion, extravascular haemorrhage, oedema, increased vascular permeability and clonogenic cell kill (Braunschweiger *et al.*, 1988).

Adrenal hormones are thought to exert a negative feedback on the immune responses (del Rey *et al.*, 1984). Naturally occurring and synthetic corticosteroid hormones are known to inhibit T cell mitogenesis (Gillis *et al.*, 1979), natural killer cell (Holbrook *et al.*, Gatti *et al.*, 1986) and macrophage (Schaffner *et al.*, 1985) function. Increases in plasma ACTH and corticosterone levels have been observed within 1 h after a single rHIL-1α treatment (Besedovsky *et al.*, 1986; del Rey *et al.*, 1987; Morrissey *et al.*, 1988), and it was postulated that such increases might exert a negative feedback on rHIL-1α stimulated immune responses (del Rey *et al.*, 1987). The possibility that this feedback pathway could be exploited, in therapeutic strategies, prompted the present studies to determine the role of adrenal hormones on rHIL-1α mediated anti-tumour activity.

### Materials and methods

### *Tumour models*

The RIF-1 fibrosarcoma tumour model was maintained according to the protocol described by Twentyman *et al.* (1980). RIF-1 cells were propagated in RPMI 1640 medium (Mediatech, Washington DC) supplemented with 10% fetal bovine serum (FBS) (Irvine Scientific, Santa Ana, CA) and 2 mM glutamine (Gibco, Grand Island, NY), as described previously (Braunschweiger *et al.*, 1982; Braunschweiger & Schiffer, 1986). RIF-1 tumours were routinely produced in 6-10 week old (~20 g), endotoxin hyposensitive, female C3H/HeJ mice (Jackson Laboratories, Bar Harbor, ME) by inoculating $5 \times 10^5$ tissue culture cells, subcutaneously, on the right flank. Studies were initiated 14 days later when tumours weighed approximately 0.5 g.

Mice were quarantined for 2 weeks before entering studies. Randomly selected mice were tested and found to be free of adventitious murine viruses. All mice were housed, 4-5 per cage, in a temperature and humidity controlled facility with a 12 h light-dark cycle (lights on at 0600 local time) accredited by the American Association for Accreditation of Laboratory Animal Care (AAALAC). Mice were provided standard mouse chow and water *ad libitum*. All procedures were reviewed and approved by the Animal Care and Use Committee of the AMC Cancer Research Center.

### *Interleukin-1α*

Recombinant human interleukin-1α was generously provided by Dr Peter Lomedico (Hoffman-LaRoche, Nutley, NJ). The rHIL-1α used in our studies was highly purified ($2.5 \times 10^9$ units $mg^{-1}$ protein, $D_{10}$ assay) and essentially free of endotoxin contamination (<0.125 EU $mg^{-1}$ protein, LAL assay). rHIL-1α was diluted in pyrogen free, sterile 0.9% NaCl containing 0.05% BSA and administered by intraperitoneal injection in 0.2 ml of vehicle. Our standard mouse dose ($6.25 \times 10^7$ $D_{10}$ units $kg^{-1}$; 25 μg $kg^{-1}$) for these studies was less than 5% of the $LD_{50}$ dose for intact mice. This dose is 2-3 times that shown by Johnson *et al.* (1989) to stimulate haematopoiesis sub-optimally in normal mice. Control mice received vehicle alone as previous studies indicated that heat inactivated rHIL-1α had no activity at this dose levels. All rHIL-1α and vehicle injections were made at 0800-0900 local time.

### *Adrenalectomy*

Bilateral adrenalectomy (ADX) was performed, via the dorsal route, using Metafane (Pittman-Moore Inc., Washington Crossing, NJ) anaesthesia, 13 days after tumour inoculation. rHIL-1α treatments were initiated on day 14. Adrenalectomised mice were given 0.9% NaCl drinking water *ad*

Correspondence: P.G. Braunschweiger.
Received 15 March 1989; and in revised form 17 July 1989.

*libitum*. In some experiments, dexamethasone (dexamethasone sodium phosphate; ESI Pharmaceuticals Inc.) was administered as $5\,mg\,kg^{-1}$ in 0.2 ml sterile, non-pyrogenic 0.9% NaCl by intraperitoneal injection.

### *Quantitative end-points*

The haemorrhagic response in RIF-1 tumours after rHIL-1α was quantitated by determining the tumour packed erythrocyte volume using a $^{59}$Fe-RBC dilution method described previously (Braunschweiger *et al.*, 1988; Braunschweiger & Schiffer, 1986). One μCi of $^{59}$Fe-citrate (NEN, Boston, MA) was injected, i.p., 7 days after tumour implantation. Studies were initiated at least 7 days later to provide adequate time for labelled erythrocytes to enter circulation and for unincorporated $^{59}$Fe to be cleared (Alpen & Cranmore. 1959). At various intervals after rHIL-1α treatment, peripheral venous blood was obtained in heparinised haematocrit tubes from the post-orbital venous plexus and centrifuged. The $^{59}$Fe radioactivity in the packed RBC fraction was determined in a gamma well counter (Packard Instruments, Downers Grove, IL) and expressed as a percentage of the injected dose (ID) per microlitre of packed RBCs (%ID $\mu l^{-1}$). After obtaining the blood sample, the mice were killed by cervical luxation. The tumours were resected *in toto* and weighed. The $^{59}$Fe radioactivity was determined, as above, and expressed as the %ID $g^{-1}$ wet weight. The tissue packed RBC volume ($\mu l\,g^{-1}$) was calculated as described previously (Braunschweiger & Schiffer, 1986).

Clonogenic cell survival in RIF-1 tumours was assessed by a modification (Braunschweiger *et al.*, 1982, 1983, 1988) of the excision clonogenic cell survival assay described originally by Twentyman *et al.* (1980), 24 h after rHIL-1α treatment. Control tumour cell yields were routinely $7-14 \times 10^7$ trypan negative cells $g^{-1}$.

### *Statistical analysis*

Analysis of variance was used to assess overall variation and the significance of post-treatment responses (Snedecor & Cochran, 1980). The Newman–Keuls multiple range test was used to determine the significance between treatment group means (Zar, 1974). Where appropriate, Student's *t* test was also used. A *P* value of less than, or equal to, 0.05 was considered adequate justification to reject the null hypothesis.

### Results

In C3H/HeJ mice untreated subcutaneous RIF-1 tumours of less than 1 g rarely exhibit surface haemorrhage, skin ulcerations, central or even local necrosis. A single intraperitoneal rHIL-1α ($6.25 \times 10^7$ units $kg^{-1}$; $25\,\mu g\,kg^{-1}$) resulted in acute haemorrhagic responses that were visually obvious within 3–6 h after treatment. Histologically, progressive vascular congestion (within 1 h) and frank vascular breakdown and haemorrhage were seen by 4 h after treatment. In ADX mice a single rHIL-1α treatment ($6.25 \times 10^7$ units $kg^{-1}$; $25\,\mu g\,kg^{-1}$) resulted in haemodynamic shock and the death of 5/5 mice by 12 h after treatment. The haemodynamic shock and systemic toxicity of rHIL-1α in ADX C3H/HeJ mice was ameliorated by giving dexamethasone ($5.0\,mg\,kg^{-1}$) within 3 h after rHIL-1α treatment. At 24 h after rHIL-1α treatment, the haemorrhagic response in RIF-1 tumours in ADX mice (dexamethasone, $5\,mg\,kg^{-1}$, 2 h after rHIL-1α) was qualitatively greater than that seen in intact mice treated with rHIL-1α alone (Figure 1). This haemorrhagic response was quantitated by the intra-tumour accumulation of $^{59}$Fe-RBC.

The effect of ADX and dexamethasone on the rHIL-1α mediated haemorrhagic responses was quantitated by determining changes in the tumour packed RBC volumes (Figure 2). Bilateral ADX was performed 24 h before rHIL-1α, and changes in tumour packed RBC volumes were determined 6 h after rHIL-1α. This study interval was chosen

**Figure 1** Representative RIF-1 tumours from intact (1a, 2a) and adrenalectomised mice (1b, 2b) 24 h after $6.25 \times 10^7$ $D_{10}$ units $kg^{-1}$ rHIL-1α (2a, 2b) or vehicle (1a, 1b). Adrenalectomised mice were given $5\,mg\,kg^{-1}$ dexamethasone 2 h after rHIL-1α to emeliorate haemodynamic shock. The adrenalectomised vehicle control (1b) also received dexamethasone. Tumours were resected 24 h after rHIL-1α treatment (48 h after adrenalectomy) and bisected along the longest axis to reveal the central region of the tumour. Control RIF-1 tumours (1a) of this size (0.5 g) rarely exhibit central necrosis or haemorrhage. In intact mice, rHIL-1α treatment resulted in significant tumour haemorrhage (2a). In adrenalectomised mice, rHIL-1α mediated haemorrhagic responses (2b) were always visually more profound than in intact mice (2a). Magnification is 1.5 ×. Bar = 1 cm.

because an equilibrium is seen by 6 h (Braunschweiger *et al.*, 1988) and, as stated above, haemodynamic toxicity after rHIL-1α in ADX mice precluded longer study intervals unless dexamethasone was also administered. Analysis of variance indicated that treatment group means were not derived from the same sample population ($F = 19.6$, $P < 0.0001$). In these studies, dexamethasone ($5\,mg\,kg^{-1}$) alone, ADX alone or ADX plus dexamethasone produced no visual haemorrhage and had no effect on tumour packed RBC volumes. rHIL-1α alone significantly ($P < 0.05$) increased tumour packed RBC volumes in both intact mice and ADX mice. The 6 h response in ADX mice was significantly ($P < 0.05$) greater than that seen in intact mice. Dexamethasone given to ADX mice, 30 min before rHIL-1α, prevented tumour haemorrhage at 6 h. When dexamethasone was given after rHIL-1α, the magnitude of the tumour haemorrhage in ADX mice (6 h) was sequence dependent. The packed RBC volumes, observed when dexamethasone was given 2 or 3 h after rHIL-1α, were greater than those seen in intact controls after rHIL-1α alone or after rHIL-1α and dexamethasone (2 h after rHIL-1α). When dexamethasone was given to ADX mice before or up to 3 h after rHIL-1α, no mortality was observed for up to 24 h after treatment. Longer observation periods were not studied.

Figure 3 shows the time dependent changes in packed RBC volumes for tumours in intact and adrenalectomised mice treated with rHIL-1α and dexamethasone ($5\,mg\,kg^{-1}$, 2 h after rHIL-1α). The haemorrhagic response in RIF-1 tumours in intact mice treated with the rHIL-1α, dexamethasone combination was not statistically different from that seen after rHIL-1α alone. However, the haemorrhagic response in tumours from adrenalectomised mice treated with the combination of rHIL-1α and dexamethasone (2 h after rHIL-1α) was greater, at all study intervals, than that seen after a similar combination in intact mice. Similar time course studies in ADX mice treated with rHIL-1α alone could not be conducted at the same rHIL-1α dose level due to severe haemodynamic toxicity.

Table I shows the results from a representative experiment to determine the effect of ADX and dexamethasone on clonogenic cell survival (24 h) in RIF-1 tumours. rHIL-1α alone resulted in 45% clonogenic cell kill. Although adrenalectomy had no significant effect on clonogenic cellularity, the combination of rHIL-1α and dexamethasone (2 h

**Figure 2** The effect of adrenalectomy (A), dexamethasone (D) and rHIL-1α ($6.25 \times 10^7$ $D_{10}$ units $kg^{-1}$) alone and in combination on packed RBC volumes of the RIF-1 tumours. Adrenalectomy was performed 24 h before rHIL-1α and packed RBC volumes were determined 6 h after rHIL-1α. Dexamethasone (5 mg $kg^{-1}$) was given 2 h after rHIL-1α in intact mice (IL-1α + D), 24 h after adrenalectomy (A + D), 30 min before rHIL-1α (A + D + IL-1α) or at 0.5, 1.0, 2.0 or 3.0 h after rHIL-1α in adrenalectomised mice. Each bar represents the mean ± 1 s.d. for six tumours. ANOVA indicated significant treatment effects and the Newman–Keuls multiple range test indicated that the response to rHIL-1α alone in intact mice was significantly different (*) from that seen in all other groups except those seen in intact mice treated with rHIL-1α + D and those in adrenalectomised mice treated with dexamethasone 30 min or 1 h after rHIL-1α.

**Figure 3** The time dependent changes in packed RBC volumes in RIF-1 tumours after rHIL-1α ($6.25 \times 10^7$ $D_{10}$ units $kg^{-1}$) and dexamethasone (5 mg $kg^{-1}$, 2 h after rHIL-1α) in intact mice (●, $n = 13$) and in mice adrenalectomised 24 h before the rHIL-1α and dexamethasone treatment (▲, $n = 6$). Also shown are data for tumours in intact mice treated with IL-1α alone (■, $n = 6$). Each symbol is the mean ± 1 s.d.

**Table I** The effect of bilateral adrenalectomy (ADX) and dexamethasone (DEX) on the survival of RIF-1 clonogenic tumour cells in representative tumours, 24 h after rHIL-1α (25 μg kg) treatment[a]

| *Treatment* | *Clonogenic cells* $g^{-1}$ $\times 10^6$ | *Surviving fraction* | $P^c$ |
|---|---|---|---|
| Control | 13.8 ± 3.2 (8)[b] | 1.00 | |
| rHIL-1α | 7.6 ± 2.2 (8) | 0.55 | <0.025 |
| ADX | 11.9 ± 3.1 (5) | 0.86 | n.s. |
| ADX + rHIL-1α + dex | 0.44 ± 0.12 (4) | 0.03 | <0.001 |

[a]Excision clonogenic cell survival assays were performed 24 h after rHIL-1α. IL-1α was administered 24 h after ADX. Dexamethasone (5 mg $kg^{-1}$) was given 2 h after rHIL-1α. Tumour tissue from three tumours was pooled for each assay.
[b]Cells were plated out *in vitro* at several dilutions in duplicate. Mean ± 1 s.d. (*n*).
[c]ANOVA and the Newman–Keuls multiple range test.

after rHIL-1α) in adrenalectomised C3H/HeJ mice resulted in approximately 97% clonogenic cell kill.

Figure 4 shows the results from a total of 26 experiments with 104 RIF-1 tumours to assess the effects of rHIL-1α alone or in combination with dexamethasone and adrenalectomy on RIF-1 clonogenic tumour cell survival. Analysis of variance indicate highly significant treatment effects ($F = 13.4$; $P < 0.0001$). The data from eight experiments indicated a 46.1 ± 4.6% (s.e.m.) surviving fraction after rHIL-1α alone (b) in intact mice. In ADX mice, the clonogenic cell survival (eight experiments) after rHIL-1α and dexamethasone (5 mg $kg^{-1}$, 2 h after rHIL-1α) was

**Figure 4** The effect of rHIL-1α ($6.25 \times 10^7$ $D_{10}$ units $kg^{-1}$) alone, or in combination with adrenalectomy and dexamethasone, on clonogenic cell survival in RIF-1 tumours. **a**, dexamethasone (5 mg $kg^{-1}$), two experiments with four tumours; **b**, rHIL-1α alone, eight experiments with 19 tumours; **c**, dexamethasone (5 mg $kg^{-1}$) given 30 min before rHIL-1α in intact mice, two experiments with four tumours; **d**, dexamethasone (5 mg $kg^{-1}$) given 2 h after rHIL-1α in intact mice, two experiments with four tumours; **e**, adrenalectomy alone, three experiments with eight tumours; **f**, dexamethasone given 30 min before rHIL-1α in adrenalectomised mice, one experiment with three tumours; **g**, dexamethasone given 2 h after rHIL-1α in adrenalectomised mice, six experiments with 14 tumours. Clonogenic cell survival assays was determined by excision 24 h after rHIL-1α or dexamethasone treatment. In each experiment, treated and control tumours were concurrently studied. Tumour tissue from two to three mice was pooled for clonogenic assays in each experiment. Each bar represents the mean ± 1 s.e. *Significantly ($P < 0.05$) different from rHIL-1α alone by ANOVA and the Newman–Keuls multiple range test.

1.27 ± 0.36% (g). This decreased survival is highly significant as compared to rHIL-1α alone ($P < 0.0005$) or the same combination (d, $P < 0.002$) in intact mice. RIF-1 clonogenic cellularity after dexamethasone (a) or adrenalectomy alone (e) was significantly greater than that seen after rHIL-1α in intact mice (b). In intact mice, rHIL-1α and dexamethasone (2 h after rHIL-1α, d) produced a surviving fraction (36.7 ± 1.4%) consistent with the additive effects of rHIL-1α and dexamethasone alone. When dexamethasone was given 30 min before rHIL-1α, clonogenic cell survival in ADX (f) and intact (c) were similar. Our results suggest that the post-rHIL-1α adrenal response not only protects the host from haemodynamic toxicity but also inhibits anti-tumour responses mediated by rHIL-1α.

### Discussion

Our data suggest that adrenal hormones play a major role in controlling rHIL-1α mediated toxicity and anti-tumour activity. Although prior ADX greatly increased haemorrhagic responses in tumours after rHIL-1α, haemodynamic toxicity was severe. Recent studies indicated that dexamethasone treatment before rHIL-1α or TNF could ameliorate the haemodynamic toxicity of these cytokines in adrenalectomised CD-1 mice (Bertini *et al.*, 1988). We have not only confirmed these findings in the C3H/HeJ mouse, but also demonstrated that dexamethasone up to 3 h after rHIL-1α prevented toxic deaths. Dexamethasone given before rHIL-1α not only prevented haemodynamic toxicity in ADX mice, but also inhibited rHIL-1α induced haemorrhagic responses in RIF-1 tumours. On the other hand, when dexamethasone was given after rHIL-1α, haemorrhagic responses increased as the sequence interval increased, up to 3 h.

Adrenalectomy not only enhanced rHIL-1α mediated vascular responses, but also increased clonogenic tumour cell kill after rHIL-1α. In intact mice, rHIL-1α alone and rHIL-1α plus dexamethasone resulted in 46% and 36% surviving fractions, respectively. We previously showed that although dexamethasone may have profound anti-proliferative effects in RIF-1 tumours (Braunschweiger *et al.*, 1982, 1983; Braunschweiger & Schiffer, 1986), dose levels much higher than those used here produced little tumour cell kill (Braunschweiger *et al.*, 1982). In the present studies, the surviving fraction after a single 5 mg kg$^{-1}$ dexamethasone treatment was 78%. Since the additive effects of the dexamethasone and rHIL-1α treatments would be expected to yield approximately 35% survival, the observed 1.27% surviving fraction for RIF-1 tumours in adrenalectomised mice treated with rHIL-1α and dexamethasone (5 mg kg$^{-1}$, 2 h after rHIL-1α; group g) would indicate that adrenal responses within 2 h after rHIL-1α have a direct or indirect antagonistic effect on rHIL-1α anti-tumour activity. This is further supported by the observation that dexamethasone given before rH-IL-1α in ADX mice (group f) inhibited rHIL-1α and mediated clonogenic cell killing.

In C3H/HeJ mice, plasma ACTH and corticosterone were increased within 1 h of rHIL-1α treatment (Besedovsky *et al.*, 1986; del Rey *et al.*, 1987; Morrissey *et al.*, 1988). Corticosteroids are known to inhibit immune functions (Gillis *et al.*, 1979; Holbrook *et al.*, 1983; Gatti *et al.*, 1986; Schaffner, 1985) and corticosteroid associated immunoregulation has been proposed as a homeostatic surveillance mechanism for the control of immune cell function (del Rey *et al.*, 1984, 1987). While this might explain the enhanced rHIL-1α antitumour responses in adrenalectomised mice, other mechanisms could be operative. rHIL-1α is known to promote the adherence of leukocytes to endothelial cells (Bevilacqua *et al.*, 1985). Since rHIL-1α can stimulate leukocytes to release proteolytic enzymes and hydrogen peroxide (Ozaki *et al.*, 1987) such responses could contribute to rHIL-1α mediated tumour vascular injuries. Further, specific high affinity glucocorticoid receptors have been demonstrated in cultured endothelial cells (Lewis *et al.*, 1986), and corticosteroids inhibit neutrophil (Ebert & Barclay, 1952), granulocyte (MacGregor, 1976) and lymphoid cell (Maca *et al.*, 1978) adherence to the endothelium.

rHIL-1α is also known to stimulate plasminogen activator inhibitor (Nachman *et al.*, 1986), procoagulant activity (Bevilacqua *et al.*, 1983) and arachidonic acid metabolism (Bernheim, 1986; Besedovsky *et al.*, 1975; Dinarello *et al.*, 1983). Prostaglandins (PG) $E_2$ and $I_2$ are potent vasodilators which can increase capillary permeability (Kuehl & Egan, 1980; Issekutz & Movat, 1983). Lipooxygenase metabolites are also vasoactive (Letts & Cirino, 1985), increase vascular permeability and promote granulocyte adherence (Samuelsson, 1983). Corticosteroids are well known inhibitors of PG and leukotriene synthesis. The concentration of hydrocortisone needed to produce near maximal inhibition of endothelial cell PG synthesis *in vitro* (Lewis *et al.*, 1986) was similar to the corticosterone concentrations measured in the sera of mice after rHIL-1α treatment (Besedovsky *et al.*, 1986; del Rey *et al.*, 1987). These findings might indicate an important role for vasoactive arachidonic acid metabolites in the rHIL-1α induced ischaemia and clonogenic cell kill in RIF-1 tumours.

The responses to rHIL-1α in RIF-1 tumours are not unlike those seen in tumours treated with tumour necrosis factor (Watanabe *et al.*, 1988; Havell *et al.*, 1988). rHIL-1α and TNF may have similar activities (Bevilaque *et al.*, 1983) and/or be synergistic (Last-Barney *et al.*, 1988; Ruggiero & Baglioni, 1987). Further, the regulation of rHIL-1α and TNF production by monocytes and macrophages may be closely interrelated (Philip & Epstein, 1986), and recent evidence suggests that these cytokines may be intimately involved in local macrophage mediated remodelling of tissue extracellular matrix (Vlassara *et al.*, 1988). Although it is tempting to postulate a role for TNF in rHIL-1α mediated tumour responses, there is as yet no direct evidence, aside from similar pathophysiologies, to support the attractive hypothesis that TNF may be a local mediator of rHIL-1α responses in RIF-1 tumours.

Since the profound vascular injury which characterised rHIL-1α responses in RIF-1 tumours was not seen in muscle and skin of intact (Braunschweiger *et al.*, 1988) or adrenalectomised hosts, the differences between tumour and normal tissue vascular structure, function and/or regulation leading to rHIL-1α induced tumour vascular injury might provide a focus for the development of new therapeutic strategies. Studies, currently underway, to investigate the negative feedback by adrenal hormones on rHIL-1α mediated tumour and host cell responses could lead to new approaches to enhance the anti-tumour activity of this multifunctional cytokine. In this regard, agents which transiently block adrenal steroid hormone synthesis (e.g. ketoconazole) potentiate rHIL-1α anti-tumour activity (Braunschweiger *et al.*, 1989) without attendant increases in toxicity.

The authors acknowledge the technical assistance of Ellen Maring and Debra Ogle and thank Kara Selby for preparation of the manuscript. This work was supported in part by Grants CA 33188, CA 33939, CA 48077 and CA 49143 from DHEW and a gift to AMC Cancer Research Center from Stephen L. Wenner.

### References

ALPEN, E. & CRANMORE, D. (1959). Cellular kinetics and iron utilization in bone marrow as observed by Fe-59 radioautography. *Ann. NY Acad. Sci.*, **77**, 753.

BERNHEIM, H.A. (1986). Is prostaglandin $E_2$ involved in the pathogenesis of fever? Effects of interleukin-1 on the release of protaglandins. *Yale J. Biol. Med.*, **59**, 151.

BERTINI, R., BIANCHI, M. & GHEZZI, P.(1988). Adrenalectomy sensitizes mice to the lethal effects of interleukin-1 and tumor necrosis factor. *Blood*, **167**, 1708.

BESEDOVSKY, H.O., DEL REY, A., SORKIN, E. & DINARELLO, C.A. (1986). Immunoregulatory feedback between interleukin-1α and glucocorticoid hormones. *Science*, **233**, 652.

BESEDOVSKY, H.O., SORKIN, E., KELLER, M. & MULLER, S. (1975). Changes in blood hormone levels during the immune response. *Proc. Soc. Exp. Biol. Med.*, **150**, 466.

BEVILAQUA, M.P., POBER, J.S., MAJEAU, G.R., FIERS, W., COTRAN, R.S. & GIMBRANE, M.A. (1983). Recombinant tumor necrosis factor induces procoagulant activity in cultured human vascular endothelium: characterization and comparison with actions of interleukin-1α. *Proc. Natl Acad. Sci. USA*, **83**, 4533.

BEVILACQUA, M.P., POBER, J.S., WHEELER, M.E., CONTRAN, R.S. & GIMBRANE, A. (1985). Interleukin-1 acts on cultured human vascular endothelium to increase the adhesion of polymorphonuclear leukocytes, monocytes and related leukocyte lines. *J. Clin. Invest.*, **76**, 2003.

BRAUNSCHWEIGER, P.G., JOHNSON, C.S., KUMAR, N., ORD, V. & FURMANSKI, P. (1988). Antitumor effects of recombinant human interleukin-1α in RIF-1 and Panc02 solid tumors. *Cancer Res.*, **48**, 6011.

BRAUNSCHWEIGER, P.G., KUMAR, N., ORD, V., JOHNSON, C.S. & FURMANSKI, P. (1989). Synergistic antitumor activity of human recombinant IL-1α and ketoconazole in RIF-1 tumors. *Proc. AACR*, **30**, 328.

BRAUNSCHWEIGER, P.G., REYNOLDS, K., NELSON, T.R. & MARING, E. (1986). The effect of dexamethasone on tissue water distribution and proton relaxation in Panc02 tumors. *Magn. Reson. Imaging*, **5**, 483.

BRAUNSCHWEIGER, P.G. & SCHIFFER, L.M. (1986). Effect of dexamethasone on vascular function in RIF-1 tumors. *Cancer Res.*, **46**, 3299.

BRAUNSCHWEIGER, P.G., TING, H.L. & SCHIFFER, L.M. (1982). Receptor dependent antiproliferative effects of corticosteroids in RIF-1 tumors and implications for sequential therapy. *Cancer Res.*, **42**, 1686.

BRAUNSCHWEIGER, P.G., TING, H.L. & SCHIFFER, L.M. (1983). Role of corticosteroid hormones in the control of cell proliferation in residual tumor after surgical cytoreduction. *Cancer Res.*, **43**, 5801.

DEL REY, A., BESEDOVSKY, H.O. & SORKIN, E. (1984). Endogenous blood levels of corticosterone control the immunologic cell mass and B-cell activity in mice. *J. Immunol.*, **133**, 572.

DEL REY, A., BESDOVSKY, H.O., SORKIN, E. & DINARELLO, C.A. (1987). Interleukin-1 and glucocorticoid hormones integrate an immunoregulatory feedback circuit. *Ann. NY Acad. Sci.*, **496**, 85.

DINARELLO, C.A, MARNOY, S.O. & ROSENWASSER, L.J. (1983). Role of arachidonic metabolism in the immunoregulatory function of human leukocytic pyrogen/lymphocyte-activating factor/interleukin-1. *J. Immunol.*, **130**, 890.

EBERT, R.H. & BARCLAY, W.R. (1952). Changes in connective tissue reaction induced by cortisone. *Ann. Intern. Med.*, **37**, 506.

GATTI, G., CAVALLO, R., SARTORI, M.L., MARINONE, C. & ANGELI, A. (1986). Cortisol at physiological concentrations and prostaglandin $E_2$ are additive inhibitors of human natural killer cell activity. *Immunopharmacology*, **11**, 119.

GILLIS, S., CRABTREE, G.R. & SMITH, K.A. (1979). Clucocorticoid induced inhibition of T-cell growth factor production I. The effect of mitogen induced lymphocyte proliferation. *J. Immunol.*, **123**, 1624.

HAVELL, E.A., FIERS, W. & NORTH, R.J. (1988). The antitumor function of murine tumor necrosis factor (TNF) I. The therapeutic action of TNF against an established murine sarcoma is indirect, immunologically dependent and limited by extreme toxicity. *J. Exp. Med.*, **167**, 1067.

HOLBROOK, N.J., COX, W.I. & HORNER, H.C. (1983). Direct suppression of natural killer activity in human peripheral blood leukocyte cultures by glucocorticords and its modulation by interferon. *Cancer Res.*, **43**, 4019.

ISSEKUTZ, A.C. & MOVAT, H.Z. (1983). The effect of vasodilator prostaglandins on polymorphonuclear leukocyte infiltration and vascular injury. *Am. J. Pathol.*, **107**, 300.

JOHNSON, C.S., KECKLER, D.J., TOPPER, M.I. BRAUNSCHWEIGER, P.G. & FURMANSKI, P. (1989). *In vitro* hematopoietic effects of recombinant interleukin-1α in mice: stimulation of granulocytic, monocytic megakaryocytic, and early erythroid progenitors, suppression of late stage erythropoiesis, and reversal of erythroid suppresion with erythropoietin. *Blood*, **73**, 678.

KUEHL, F.A. & EGAN, R.W. (1980). Prostaglandins, arachidonic acid and inflammation. *Science*, **210**, 978.

LACHMAN, L.B., DINARELLO, C.A., LLANSA, N.D. & FIDLER, I.J. (1986). Natural and recombinant human interleukin 1α is cytotoxic for human melanoma cells. *J. Immunol.*, **136**, 3098.

LAST-BARNEY, L., HOMON, C.A., FAANES, R.B. & MERLUZZI, V.J. (1988). Synergistic and overlapping activities of tumor necrosis factor-α and IL-1. *J. Immunol.*, **141**, 527.

LETTS, L.G. & CIRINO, M. (1985). Vascular actions of leukotrienes. *Prog. Clin. Biol. Res.*, **199**, 47.

LEWIS, G.D., CAMPBELL, W.B. & JOHNSON, A.R. (1986). Inhibition of prostaglandin synthesis by glucocorticoids in human endothelial cells. *Endocrinology*, **119**, 62.

MACA, R.D., FRY, G.L. & HAKES, A.D. (1978). Effects of glucocorticoids on the interaction of lymphoblastoid cells with human endothelial cells *in vitro*. *Cancer Res.*, **38**, 2224.

MACGREGOR, R.R. (1976). The effect of anti-inflammatory agents and inflammation on granulocyte adherence. Evidence for regulation by plasma factors. *Am. J. Med.*, **61**, 597.

MORRISSEY, P.J., CHARRIER, K., ALPERT, A. & BRESSLER, L. (1988). *In vivo* administration of IL-1 induces thymic hypoplasia and increased levels of serum corticosterone. *J. Immunol.*, **141**, 1456.

NACHMAN, R.L., HAJJAR, K.A., SILVERSTEIN, R.L. & DINARELLO, C.A. (1986). Interleukin-1 induces endothelial cell synthesis of plasminogen activator inhibitor. *J. Exp. Med.*, **163**, 1595.

NAKAMURA, S., NAKATA, K., KASHIMOTO, S., YOSHIDA, H. & YAMADA, M. (1986). Antitumor effect of recombinant interleukin alpha against murine syngeneic tumors. *Jpn. J. Cancer Res. (Gann)*, **77**, 767.

NAKATA, K., KASHIMOTO, S., YOSHIDA, H., OKU, T. & NAKAMURA, S. (1988). Augmented antitumor effect of recombinant human interleukin-1 by indomethacin. *Cancer Res.*, **48**, 584.

OZAKI, Y., OHASHI, T. & KUME, S. (1987). Potentiation of neutrophil function by recombinant DNA-produced interleukin-1α *J. Leuko. Biol.*, **42**, 621.

PHILIP, R. & EPSTEIN, L.B. (1986). Tumor necrosis factor as immunodulator and mediator of monocyte cytotoxicity induced by itself, gamma-interferon and interleukin 1. *Nature*, **323**, 86.

RUGGIERO, V. & BAGLIONI, C. (1987). Synergistic antiproliferative activity of Interleukin-1 and tumor necrosis factor. *J. Immunol.*, **138**, 661.

SAMUELSSON, B. (1983). Leukotrienes: mediators of immediate hyper sensitivity reactions and inflammation. *Science*, **220**, 568.

SCHAFFNER, A. (1985). Therapeutic concentration os glucocorticords suppress the antimicrobial activity of human macrophages without impairing their responsiveness to gamma interferon. *J. Clin. Invest.*, **76**, 1755.

SNEDECOR, G.W. & COCHRAN, W.G. (1980). *Statistical Methods*. Ames, IA: Iowa State University Press.

TWENTYMAN, P.R., BROWN, J.M., GRAY, J.W., FRANK, A.J., SCOLES, M.A. & KALLMAN, R.F. (1980). A new mouse tumor model system (RIF-1) for comparison of endpoints. *J. Natl Cancer Inst.*, **64**, 595.

VLASSARA, H., BROWNLEE, M., MANOGUE, K.R., DINARELLO, C.A. & PASAGIAN, A. (1988). Cachectin/TNF and Il-1 induced by glucose-modified proteins: role in normal tissue remodeling. *Science*, **240**, 1546.

WATANABE, N., NIITSU, Y., UMENO, H. & 5 others (1988). Toxic effect of tumor necrosis factor on tumor vasculature in mice. *Cancer Res.*, **48**, 2179.

ZAR, J.H. (1974). *Biostatistical Analysis*. Englewood Cliffs, NJ: Prentice Hall.